# Tracking multidrug-resistant bacteria: a one-year retrospective study at Moulay Ismail Military Hospital, Meknes, Morocco

**DOI:** 10.11604/pamj.2026.53.53.46786

**Published:** 2026-02-04

**Authors:** Reda Karami, Rabii El Bahraouy, Mourad Belaouni, Mohammed Er-Rami, Lhoussain Louzi, Mohammed Sbiti

**Affiliations:** 1 Microbiology Laboratory, Moulay Ismail Military Hospital, Meknes, Morocco,; 2 Sidi Mohamed Ben Abdellah University, Faculty of Medicine, Pharmacy and Dentistry, Fez, Morocco,; 3 Parasitology-Mycology Laboratory, Moulay Ismail Military Hospital, Meknes, Morocco,; 4 Mohammed V University, Faculty of Medicine and Pharmacy, Rabat, Morocco

**Keywords:** Antimicrobial resistance, antibiograms, multidrug-resistant organisms, drug resistance, anti-bacterial agents

## Abstract

**Introduction:**

antimicrobial resistance represents a major global public health concern, fueled by the growing prevalence of multidrug-resistant bacteria that limit therapeutic options. This study aims to describe the epidemiological characteristics of multidrug-resistant bacteria isolated in our facility and to evaluate their antibiotic resistance profiles.

**Methods:**

this is a retrospective and descriptive study covering one year (June 2022 to May 2023), examining the results of antibiograms performed at the bacteriology laboratory of Moulay Ismail Military Hospital in Meknes, Morocco.

**Results:**

we examined 1217 bacterial strains and found that 23.5% were multidrug-resistant. ESBL-E comprises 51% of these multidrug-resistant bacteria, followed by 6% MRSA, 19% IRAB, and 19% CPE. The intensive care unit serves as the primary source of isolation for multidrug-resistant bacteria (36%), followed by surgical services (27%). Urine is the primary reservoir for these bacteria (55%). The analysis of the resistance profile of these multidrug-resistant bacteria demonstrated a notable prevalence of resistance to various classes of antibiotics. Fortunately, most of these multidrug-resistant bacteria remain susceptible to amikacin and fosfomycin, exhibiting complete sensitivity to colistin.

**Conclusion:**

enhancing antibiotic prescribing practices and strictly implementing hygiene and preventative protocols would aid in reducing the development of multidrug-resistant organisms. An in-depth understanding of bacterial ecology and resistance profiles to various antibiotics would provide customized treatment for each medical setting.

## Introduction

Antimicrobial resistance today represents a substantial danger to worldwide public health. Since the initial decades of antibiotic use, numerous pathogenic bacteria have evolved resistance mechanisms, undermining treatment efficacy and complicating infection management. Despite advancements since Alexander Fleming's discovery of penicillin in 1928, the excessive and improper use of antibiotics has expedited the rise of multidrug-resistant microorganisms. Microorganisms that are resistant to several antibiotic classes are hard to treat in hospitals, where selection pressure helps them spread through mobile genetic elements [1,2].

The ramifications are both medical and economic. Multidrug-resistant (MDR) bacterial infections extend hospitalizations, resulting in elevated expenses and care requirements that often necessitate last-resort therapies that are typically more hazardous, costly, and less efficacious [3]. The World Health Organization (WHO) reports that bacterial resistance has reached critical levels in multiple countries, potentially exceeding cancer as the foremost cause of worldwide mortality by 2050, with an estimated almost 10 million fatalities annually [4]. Despite the lack of comprehensive data, research from university hospitals in Morocco reveals a concerning situation. In 2019, the WHO partnered with Morocco to formulate a national strategy to address antimicrobial resistance (AMR), employing a “One Health” framework that integrates human, animal, and environmental health [5].

This study seeks to provide an overview of the MDR bacteria isolated in the bacteriology laboratory at Moulay Ismail Military Hospital in Meknes over a twelve-month period. This document analyzes the epidemiological profile of the bacteria, assesses their antibiotic resistances, and offers data that may inform clinical practices about antibiotic prescriptions for community-acquired and nosocomial illnesses.

## Methods

**Study design:** this descriptive and retrospective study was conducted over a period of twelve months (June 2022 to May 2023) at the bacteriology laboratory of the Meknes-based Moulay Ismail Military Hospital, a 350-bed healthcare facility providing several specialties, excluding pediatrics.

**Study group:** this investigation encompassed all bacteriological samples designated for diagnostic analysis, irrespective of their origin from hospitalized patients or external consultants. The exclusion criteria included bacterial isolates from the same patient exhibiting the same sensitivity profile (duplicates) and environmental samples.

**Laboratory procedure:** the gathered samples underwent a standardized analysis tailored to each sample type. This analysis includes a comprehensive macroscopic evaluation. It involves wet mount assessment, Gram staining for bacterial differentiation and classification, bacterial culture using specialized media suited to sample characteristics, bacterial identification through biochemical and culture assays, API® for microorganism identification (BioMérieux, France), chromogenic media, and the BD Phoenix™ M50 system (Becton Dickinson, United States).

**Antibiogram:** we used the Mueller-Hinton agar medium diffusion technique and followed the rules set by the Antibiogram Committee of the French Society of Microbiology (CA-SFM) and the European Committee on Antimicrobial Susceptibility Testing (EUCAST) in their January 2019 edition to test how sensitive bacteria were to antibiotics. We also utilized the automated antibiogram method with the BD Phoenix™ M50 to determine the minimum inhibitory concentrations (MIC).

**Identification of multidrug-resistant bacteria:** Extended-spectrum β-lactamase-producing *Enterobacteriaceae* (ESBL-E): we used the synergy assessment to identify ESBL production by detecting the champagne-cork phenomenon. We positioned an amoxicillin plus clavulanic acid disk in the center of the petri dish, 30mm away from four disks containing cefotaxime, ceftazidime, cefepime, and aztreonam.

***Carbapenemase-producing Enterobacteriaceae (CPE):*** we used the CA-SFM/EUCAST (January 2019) algorithm and found the type of carbapenemase using a fast immunochromatographic assay (Coris BioConcept, Belgium).

***Methicillin-resistant Staphylococcus aureus (MRSA):*** requires a cefoxitin susceptibility test with a diameter of less than 22mm on Mueller-Hinton agar medium.

***Resistance of imipenem-resistant Acinetobacter baumannii (IRAB), imipenem-resistant Pseudomonas aeruginosa (IRPA), and carbapenem-resistant Pseudomonas aeruginosa (CRPA):*** this was evaluated using imipenem and ceftazidime disks, following CA-SFM/EUCAST guidelines (January 2019).

**Statistical analysis:** the collected data was analyzed using Jamovi software version 2.6 [6]. The overall and specific prevalences of MDR bacteria were calculated, together with the distribution frequencies of isolates classified by hospital departments, sample types, and resistance profiles. A comparative statistical analysis with the chi-square test (χ^2^) for qualitative variables to compare groups, including hospitalized patients versus external consultants, hospital departments, and types of isolates. A p-value of 0.05 or less was considered statistically significant.

## Results

We examined 1217 samples, comprising 75% from males and 25% from women, resulting in a male-to-female sex ratio of 3: 1. Of the isolated bacteria, 70% were enterobacteria, 17% were *Staphylococci*, 8% were *Pseudomonas aeruginosa*, and 5% were *Acinetobacter baumannii*. The antibiogram identified 286 MDR bacteria, reflecting a total prevalence of 23.5%. The ESBL-E constituted over fifty percent of the MDR bacteria. The remaining categories were allocated as follows: IRAB and CPE at 19% each, MRSA at 6%, CRPA at 4%, and IRPA at 1% (Table 1).

**Table 1:** distribution of multidrug-resistant bacteria by isolates

Isolates (n = 1217)	MDR phenotype (n = 286)
Enterobacteria (n = 851)	ESBL-E (n = 145)
	CPE (n = 54)
*Staphylococci* (n = 209)	MRSA (n = 16)
*Pseudomonas aeruginosa* (n = 98)	CRPA (n = 12)
	IRPA (n = 4)
*Acinetobacter baumannii* (n = 59)	IRAB (n = 55)

MDR: multidrug-resistant. ESBL-E, extended-spectrum beta-lactamase *Enterobacteriaceae*. CPE, carbapenemase-producing *Enterobacteriaceae*. MRSA, methicillin-resistant *Staphylococcus aureus*. CRPA, carbapenem-resistant *Pseudomonas aeruginosa*. IRPA, imipenem-resistant *Pseudomonas aeruginosa*. IRAB, imipenem-resistant *Acinetobacter baumannii*

Hospitalized patients had a considerably greater prevalence of MDR bacteria (67.5%, n=193) than outpatient patients (32.5%, n=93). All MDR bacterial groups, especially IRAB and CRE, exhibited this tendency. Among the ESBL-E, *Escherichia coli* constituted 58% of the isolates, followed by *Klebsiella pneumonia* at 19%, which was also the most prevalent among the CRE, totaling 44%. Urine samples were the primary source of MDR bacteria (55%), followed by respiratory samples (17%), pus samples (15%), and blood cultures (8%) (Table 2).

**Table 2:** distribution of multidrug-resistant bacteria per samples

Sample category (n = 286)	ESBL-E (n = 145)	IRAB (n = 55)	CPE (n = 54)	MRSA (n = 16)	CRPA (n = 12)	IRPA (n = 4)
CBEU (n = 156)	105	10	27	7	4	3
Pus samples (n = 43)	18	8	11	2	3	1
Respiratory specimens (n = 49)	10	32	4	1	2	0
Blood cultures (n = 23)	5	3	9	3	3	0
Puncture fluids (n = 8)	5	1	1	1	0	0
Other samples (n = 7)	2	1	2	2	0	0

CBEU, cyto-bacteriological examination of urine. ESBL-E, extended-spectrum beta-lactamase *Enterobacteriaceae*. IRAB, imipenem-resistant *Acinetobacter baumannii*. CPE, carbapenemase-producing *Enterobacteriaceae*. MRSA, methicillin-resistant *Staphylococcus aureus*. CRPA, carbapenem-resistant *Pseudomonas aeruginosa*. IRPA, imipenem-resistant *Pseudomonas aeruginosa*.

Among the 193 MDR bacteria identified from hospitalized patients, the primary source was the intensive care units (36%), followed by the surgical and medical services (27% each). The emergency department represented 10% of the isolates. The ESBL-E and CPE predominantly originated from the surgical services (28/52 and 15/52, respectively) and medical services (26/52 and 12/52, respectively). Intensive care prominently represented the IRAB (42/69), while surgery (3/52) and medicine (4/52) showed a comparable distribution of MRSA (Table 3). The urology department was the primary reservoir of MDR bacteria within the surgical services (22/52), followed by the visceral surgery department (10/52) (Table 4).

**Table 3:** distribution of multidrug-resistant bacteria by hospital departments

Departments (n = 193)	ESBL-E (n = 85)	IRAB (n = 51)	CPE (n = 37)	MRSA (n = 11)	CRPA (n = 8)	IRPA (n = 1)
Intensive care unit (n = 69)	12	42	9	4	2	0
Surgical services (n = 52)	28	4	15	3	2	0
Medical services (n = 52)	26	5	12	4	4	1
Emergency (n = 20)	19	0	1	0	0	0

ESBL-E, extended-spectrum beta-lactamase *Enterobacteriaceae*. IRAB, imipenem-resistant *Acinetobacter baumannii*. CPE, carbapenemase-producing *Enterobacteriaceae*. MRSA, methicillin-resistant *Staphylococcus aureus*. CRPA, carbapenem-resistant *Pseudomonas aeruginosa*. IRPA, imipenem-resistant *Pseudomonas aeruginosa*.

**Table 4:** distribution of multidrug-resistant bacteria by surgical services

Surgical services (n = 52)	Proportion (%)
Urology (n = 22)	42
Visceral Surgery (n = 10)	19
Traumatology and Orthopedics (n = 5)	10
Gynecology (n = 4)	8
Thoracic surgery (n = 3)	6
Plastic surgery (n = 3)	6
ENT (n = 3)	6
Neurosurgery (n = 2)	4

The examination of the resistance profiles of the 286 isolated MDR bacteria indicates alarming trends in antibiotic resistance. Four percent of ESBL-E (n=145) displayed resistance to imipenem, five percent to fosfomycin, and one isolate (0.7%) showed resistance to colistin. MRSA (n=16) showed full susceptibility to vancomycin and teicoplanin, but significant resistances were noted against ciprofloxacin (13%), erythromycin (31%), and fusidic acid (44%). In CPE (n=54), there was significant resistance to fluoroquinolones (70%), gentamicin (96%), and sulfamethoxazole plus trimethoprim (96%), although colistin demonstrated complete efficacy. The immunochromatographic analysis indicated that 41% of carbapenemases are of the NDM type, 26% are of the OXA-48 type, and 15% are of the NDM+OXA-48 type. IRAB (n=55) displayed significant multi-resistance, showing total resistance to carbapenems, cephalosporins, and ciprofloxacin. The resistance to aminoglycosides, specifically amikacin and gentamicin, was 82% and 91%, respectively, although colistin demonstrated complete efficacy. CRPA (n=12) exhibited substantial resistance to gentamicin (67%), levofloxacin (58%), and ciprofloxacin (58%), while displaying complete sensitivity to colistin. IRPA (n=4) demonstrates sensitivity to most antibiotics.

## Discussion

**The increasing prevalence of multidrug-resistant bacteria is alarming:** the analysis revealed a prevalence of MDR bacteria at 23.5%, highlighting a notable increase on both national and international levels. Documentation confirms the following prevalence rates in Morocco: 11.8% in Fez in 2012 [7], 16% in Marrakech in 2016 [8], 24% in Oujda in 2018 [9], and 32% in Marrakech in 2019 [10]. Globally, we observed 33.4% in China in 2012 [11], 35% in Italy in 2020 [12], and 26.6% in Tunisia in 2020 [13]. Socioeconomic conditions, antibiotic use legislation, and medication availability influence variations at both regional and national levels [14]. The notable increase in prevalence serves as a powerful call to action, highlighting the urgent need for measures to tackle this issue.

**Most of our multidrug-resistant bacteria are ESBL-E:** ESBL-E accounts for 51% of all MDR bacteria, consistent with national and international data trends. In 2016, the figure was 63% in Marrakech [8], 63% in Rabat in 2012 [15], 59.5% in Monastir in 2020 [13], and 45% in China in 2012 [11].

***IRAB (19%):*** while its prevalence in our study is comparatively low, it continues to be a significant concern in other contexts. In 2012, the percentage was 39% in Fez [7]; in 2016, it was 26% in Marrakech [8]; and in 2012, it was 23% in China [11]. In 2020, the rate in Italy was 2.4% [12].

***CPE (19%):*** a substantial increase has been observed over the years. In Morocco, the increase in Marrakech rose from 2.3% in 2016 [8] to 16% in 2019 [10]. In 2020, the international rates were 10.5% in Monastir [13] and 26% in Italy [12].

***MRSA (6%):*** this figure constitutes a minority in our study, consistent with national trends: 2% in Rabat in 2012 [15], 7% in Marrakech in 2016 [8], and 8% in Monastir in 2020 [13]. Conversely, Italy exhibited a significant rate of 31% in 2020 [12]. Multidrug-resistant *Pseudomonas aeruginosa* has shown temporal stability, consistently occupying the lowest position in the distribution of MDR bacteria (Table 5). Table 5 illustrates the significant public health challenges posed by ESBL-E and IRAB at both national and international levels. Several things make this problem worse: overusing β-lactams, insufficient infection control strategies, the fast spread of resistance genes, and the basic molecular mechanisms at play. IRAB strains are notably characterized by their rapid spread in hospital environments, attributed to their resistance to disinfectants and their capacity to endure on surfaces. The presence of these characteristics leads to a heightened dissemination of MDR bacteria within hospital settings and, in certain instances, extends to surrounding communities.

**Table 5:** comparison of our isolated MDR bacteria with national and international literature

Study	ESBL-E %	IRAB %	CPE %	MRSA %	CRPA %	IRPA %
Fez 2012 [7]	47	39	NS	4	9	NS
Rabat 2012 [15]	63	26,5	NS	2	8,5	NS
Marrakech 2016 [8]	63	26	2,3	7	1,7	NS
Marrakech 2019 [10]	45	25	16	7	4	3
Monastir 2020 [13]	59,5	13	10,5	8	2	7
China 2012 [11]	45	23,3	1,3	13	17,7	NS
Italy 2020 [12]	36	2,4	26	31	4,6	NS
Meknes 2023 (our study)	51	19	19	6	4	1

NS, not studied. ESBL-E, extended-spectrum beta-lactamase *Enterobacteriaceae*. IRAB, imipenem-resistant *Acinetobacter baumannii*. CPE, carbapenemase-producing *Enterobacteriaceae*. MRSA, methicillin-resistant *Staphylococcus aureus*. CRPA, carbapenem-resistant *Pseudomonas aeruginosa*. IRPA, imipenem-resistant *Pseudomonas aeruginosa*

Our results clearly show that *Escherichia coli* is common among MDR bacteria, especially among those that make extended-spectrum beta-lactamase. This is in line with many other national and international research studies. For instance, researchers have documented comparable rates in Fez (49% in 2012) [7], Marrakech (44.2% in 2016) [8], and Rabat (42% in 2020) [16]. However, the 2019 study in Marrakech presents an important exception, revealing that *Klebsiella spp*. was the most prevalent, accounting for 42.5%, followed by *Escherichia coli* at 22.5% [10]. Global research has documented the dominance of *Escherichia coli* among ESBL-E, with reported proportions ranging from 41% in Monastir [13] to 71.7% in China [11]. *Escherichia coli's* exceptional adaptive capabilities, which facilitate the acquisition and dissemination of resistance genes via mechanisms like conjugation, plasmid integration, or genetic mutation, contribute to its prevalence. The processes involved, especially the production of extended-spectrum β-lactamases (ESBLs) through plasmids [17], make it much easier for bacteria to live in a variety of environments and spread these resistances. This may explain why we saw such a high frequency in our results.

The results of this study indicate that *Klebsiella spp*. is the most prevalent species among the CRE, accounting for 60% of the isolates, which aligns with findings from both national and international literature [14,18]. The exceptional capability of *Klebsiella spp*. to generate carbapenemases and obtain resistance genes via genetic mechanisms like plasmid transfer explains its dominance. *Klebsiella spp*. is also very easily spread in healthcare settings, especially among people who don't have strong immune systems. This is a big reason why it is becoming more and more common to get sick in hospitals [19].

Our results indicated a higher prevalence of male patients among those diagnosed with MDR bacteria. Additionally, we observed a statistically significant correlation between sex and the overall prevalence of MDR bacteria, suggesting a higher incidence in men than women. This observation aligns with existing literature, which typically indicates a male-to-female sex ratio greater than 1. Nonetheless, our sex ratio markedly exceeds that observed in most studies. The nearest findings are those documented in the military hospitals of Rabat (sex ratio of 2.57) [15] and Marrakech (1.96) [8]. The unique nature of these facilities, primarily serving male patients, accounts for this. The differences in the sex ratio noted across various studies may indicate regional or institutional variations, especially in the prescribing practices for antibiotics, such as those used for urinary infections like cystitis, which predominantly impact women. The variations underscore the necessity of considering demographic and institutional contexts when examining epidemiological data.

Our findings indicate that 67.5% of the isolated MDR bacteria originate from hospitalized patients, with significant numbers observed in the intensive care unit (35%). We found a statistically significant link between the types of MDR bacteria and hospital departments. The most common strains were IRAB in the intensive care unit and ESBL-E and CPE in the medical and surgical services. This finding aligns with a broader national trend, indicating that intensive care units are consistently the most impacted by MDR bacteria, followed by surgical services and then medical services. This distribution shows how intensive care units work. Long hospital stays, immunocompromised patients, the common use of invasive devices like tubes and catheters, and multiple antibiotic treatments all greatly increase the chance of getting an infection from bacteria that are resistant to multiple antibiotics [20]. It is noteworthy that, in an Indian study [21], surgical services are the predominant focus. Inadequate preventive measures involving broad-spectrum antibiotics [22], the severity of invasive interventions, the application of synthetic materials, and the use of inappropriate prosthetic devices [23] contribute to this specific characteristic. These observations underscore the critical need for effective antibiotic management and clinical practices to mitigate the rise of MDR bacteria in diverse hospital environments.

According to the findings, urine cultures yielded a significant proportion of MDR bacteria (54.5%), supporting the idea that urine is the primary source for isolating MDR bacteria, which aligns with national research. However, the Marrakech investigation in 2019 stands out as an important exception, with pus samples being the most prevalent, accounting for 34.7% [10]. Additionally, respiratory samples constitute a significant portion of our study, likely due to the elevated occurrence of IRAB infections, which are frequently linked to invasive devices in intensive care settings. Furthermore, we identified a notable correlation between the various sample categories and the source of the MDR bacteria, which could be either hospitalized or external patients. External patients were the primary source of urine samples, while hospitalized patients, particularly those in intensive care, provided almost all respiratory samples. Globally, blood cultures often serve as the main source for isolating MDR bacteria, with findings from Qatar indicating a rate of 27.5% and Senegal reporting 27% [24,25]. It is crucial to recognize that these studies concentrated solely on ESBL-E and did not encompass all MDR bacteria, which could account for this discrepancy. The findings underscore the urinary tract as a significant reservoir for MDR bacteria, including ESBL-E, CPE, MRSA, and others. The primary risk factor for hospital-acquired urinary infections caused by MDR bacteria continues to be urinary catheterization. This underscores the importance of having stringent guidelines and minimizing the use of these devices to avert colonization and related infections [26]. Our study's respiratory samples show that imipenem-resistant *Acinetobacter baumannii* (IRAB) is very common. It makes up 65% of the respiratory isolates and is mostly found in intensive care units. Various factors unique to intensive care units can explain this overrepresentation. To begin with, *Acinetobacter baumannii* demonstrates a remarkable capacity to endure extended durations within the hospital setting, facilitating its persistence and spread. Subsequently, the delicate condition of patients in intensive care, frequently with compromised immune systems, along with extended hospitalizations, heightens their susceptibility to this pathogen. Ultimately, mechanical ventilation procedures, especially intubation, are crucial in establishing a pathway for the infection.

The observations underscore the critical need for meticulous oversight of care practices within intensive care units. This includes strict hygiene rules, limiting the length of intubation, and improving the care of invasive devices to stop the spread and colonization of *A. baumannii*.

**Resistance characteristics of ESBL-producing *Enterobacteriaceae*:** isolated ESBL-E exhibit distinct resistance rates to aminoglycosides, with 63% resistance to gentamicin and 7% resistance to amikacin. The results align with findings from Meknes in 2015 (61%) [27] and Qatar in 2016 (67%) [24], yet are lower than the rates observed in Algeria (90%) [28], Lebanon (78%) [23], and Marrakech (85%) [29]. The resistance rates to amikacin are low, at 7% in Rabat [16] and 5% in Marrakech [29], indicating its potential as a therapeutic option, although data from Algeria shows a concerning rate of 56% [28]. The elevated resistance rate to ciprofloxacin among fluoroquinolones, recorded at 93%, is a significant concern. The prevalence associated with the transmission of resistance genes, particularly the qnr gene [30], indicates a concerning trend: 80% in Marrakech [29], 87% in Rabat [16], and 90% in Algeria [28]. The extensive and unregulated application of molecules such as ciprofloxacin in urinary infections significantly exacerbates this critical situation.

Our study indicates a low resistance level to fosfomycin at 5%, consistent with findings from Rabat (8%) [16], Meknes (6%) [27], and Lebanon (6%) [23]. However, these results sharply contrast with those from Marrakech (74%) [29], where more frequent use appears to explain increased resistance. The unavailability of fosfomycin in Morocco may account for its limited use, thereby positioning it as a promising alternative. Carbapenems, especially imipenem, demonstrate strong efficacy, exhibiting only 4% resistance in our study, comparable to findings from Marrakech (7%) [29] and Meknes (6%) [27]. However, the consistent observation of total sensitivity, as reported in Algeria [28] and Lebanon [23], has faded, requiring increased vigilance due to increasing resistance, particularly in Rabat (16%) [16].

In conclusion, the detection of a single colistin-resistant strain, although limited, is a matter of concern. Resistance to colistin through plasmids may make it less effective, especially in intensive care units where it is often one of the last therapeutic options left. These observations underscore an increasing constraint on therapeutic options in the context of ESBL-E infections. The increase in resistance, especially to carbapenems, highlights the necessity for revising therapeutic strategies. Appropriate use of molecules like fosfomycin and amikacin, along with organized management of antibiotics, is important to keep treatment options open and prevent the growth of resistance.

**Resistance characteristics of carbapenemase-producing *Enterobacteriaceae*:** the results show that the CPE exhibits a significantly elevated resistance rate to aminoglycosides, surpassing the levels documented in Rabat in 2014 [31] and in Algeria in 2017 [32]. Tunisia conducted a study in 2016 that revealed nearly complete resistance to gentamicin and a 51% resistance rate to amikacin [33]. In our study, we observed a resistance to imipenem of 96%, which surpasses the reported resistance in comparative studies. Our findings regarding fluoroquinolones align with existing literature, except for Algeria, which indicates a lower resistance rate to ciprofloxacin at 17%. Our findings on carbapenemase types align with those reported in Tunisia in 2017 [34], showing rates of NDM (59%), OXA-48 (33%), and NDM + OXA-48 (8%). The multidrug resistance seen is probably because of using too many carbapenems and the fact that carbapenemase genes often occur together with other resistance genes [35]. The exploration of alternative strategies, especially antibiotic combinations, is crucial for reducing morbidity and mortality as well as the emergence of resistance [36,37].

**Resistance characteristics of methicillin-resistant *Staphylococcus aureus*:** the results obtained for MRSA in our study align with findings from Marrakech (2022) [38] and Fes (2013) [7], with the latter study reporting a notably high resistance to fusidic acid at 61%. The resistance profiles in Saudi Arabia correspond with our findings [39], whereas in India, there is a reported total resistance to fluoroquinolones, aminoglycosides, and macrolides [40]. Despite this variability, one constant persists: the total sensitivity of MRSA to glycopeptides, which remain effective irrespective of resistance to other antibiotics. These molecules are crucial in the management of severe MRSA infections.

**Resistance characteristics of imipenem-resistant *Acinetobacter baumannii*:** in this study, all *Acinetobacter baumannii* strains exhibited sensitivity to colistin, consistent with local data. Notably, the resistance to ceftazidime in our study is 100%, in contrast to 66% reported in Marrakech in 2022 [38]. Comparable findings from a 2014 Brazilian study show a notable difference in gentamicin efficacy (76.6% versus 90% in our study) [41]. The decreasing effectiveness of carbapenems is concerning, as they frequently serve as the final line of defense against *A. baumannii*, a pathogen with numerous natural and acquired resistances [42]. Colistin retains efficacy; however, its application requires stringent regulation owing to significant adverse effects.

**Resistance characteristics of *Pseudomonas aeruginosa*:** the *Pseudomonas aeruginosa* isolates examined in this study exhibit extensive resistance to multiple antibiotic classes, indicating significant variability across the compared studies. Colistin maintains its effectiveness against these strains, except for a Spanish study that reports a 5.4% resistance rate [43]. The severity of *Pseudomonas aeruginosa* infections depends on the bacterium's intrinsic virulence, its capacity to develop resistance mechanisms, and patient comorbidities, which limit the treatment options. This underscores the necessity for targeted strategies to prevent the emergence of resistant strains [44].

**Limitations:** since this is a retrospective study, making associations between trends in antibiotic resistance and clinical outcome will be impossible. Prospective studies would define risk factors for the infection better and the outcomes of treatment more appropriately. Our study was performed in only one health facility; hence, it might not fully represent other hospitals either in Morocco or in other different settings. Different infection control practices, antibiotic stewardship programs, and patient populations might be reasons for dissimilar resistance patterns. Resistance traits to various drugs were detected by phenotypic techniques but not verified by molecular approaches (e.g., PCR or whole genome sequencing) to characterize exact resistances or clonality. This limits the ability to determine the specific genetic mechanisms underlying resistance trends. This investigation focused primarily on microbiological data without traditional patient-centered clinical outcomes, such as treatment success, morbidity, or mortality. Readers would benefit from a correlation of resistance patterns with clinical prognosis—an association that would increase the clinical relevance of the study. Our study concentrated on hospital-acquired infections and community-acquired multidrug-resistant bacteria; however, it did not investigate environmental reservoirs. Future studies should use a “One Health” framework to evaluate the comprehensive dynamics of resistance dissemination.

## Conclusion

Antibiotic resistance poses a considerable threat to global public health, resulting in extended hospital stays, elevated medical costs, and increased rates of morbidity and mortality. An in-depth understanding of the local bacterial ecology is crucial for formulating suitable and effective strategies. This study, carried out over twelve months at the Meknes-based Moulay Ismail Military Hospital, emphasizes the epidemiological profile and resistance trends of MDR bacteria. ESBL-E, MRSA, and CPE are significant concerns, especially within intensive care and surgical services. Because the bacteria are resistant to many drugs, there aren't as many therapeutic options. Molecular options include fosfomycin, amikacin and colistin. The risk of a therapeutic impasse significantly increases due to the rapid proliferation of these bacteria and the lack of new promising molecules. Addressing this challenge necessitates the preservation of the efficacy of existing antibiotics, which demands careful, rational, and targeted application. In conclusion, continuous development and updating of bacterial resistance surveillance programs are essential at both local and national levels. The Committees for the Fight Against Nosocomial Infections play a crucial role in preventing and controlling this issue, facilitating the dynamic adaptation of therapeutic and prophylactic protocols.

### What is known about this topic

- Multidrug-resistant bacteria are a significant public health concern contributing to morbidity, mortality, and healthcare costs; overuse of antibiotics, poor infection control, and the spread of resistant strains within healthcare settings all contribute to resistance developing;
- The most important multidrug-resistant pathogens are Klebsiella pneumoniae, Escherichia coli, Pseudomonas aeruginosa, Acinetobacter baumannii and Staphylococcus aureus; they are usually resistant to antibiotics, including carbapenemases (KPC, NDM), extended-spectrum beta-lactamases, and methicillin;
- Implementing effective surveillance programs, promotion of antimicrobial stewardship, and strict enforcement of infection control measures (including hand hygiene and isolation protocols) are crucial strategies to control their spread.

### What this study adds

- Our data provide local epidemiological relevant information on the burden, distribution, and resistance patterns of MDR bacteria in a specific Moroccan healthcare setting, which has been lacking in the regional epidemiological data;
- Our work identifies the most frequently isolated MDR pathogens at our facility, including resistance mechanisms and potential risk factors; these findings contribute to the development of targeted infection control measures and antimicrobial stewardship strategies.
